# Identification of Subtypes and a Prognostic Gene Signature in Colon Cancer Using Cell Differentiation Trajectories

**DOI:** 10.3389/fcell.2021.705537

**Published:** 2021-12-13

**Authors:** Renshen Xiang, Jincheng Fu, Yuhang Ge, Jun Ren, Wei Song, Tao Fu

**Affiliations:** ^1^ Department of Gastrointestinal Surgery II, Renmin Hospital of Wuhan University, Wuhan, China; ^2^ Central Laboratory, Renmin Hospital of Wuhan University, Wuhan, China

**Keywords:** colon cancer, cell differentiation trajectory, immunotherapy, chemotherapy, risk scoring

## Abstract

Research on the heterogeneity of colon cancer (CC) cells is limited. This study aimed to explore the CC cell differentiation trajectory and its clinical implication and to construct a prognostic risk scoring (RS) signature based on CC differentiation-related genes (CDRGs). Cell trajectory analysis was conducted on the GSE148345 dataset, and CDRG-based molecular subtypes were identified from the GSE39582 dataset. A CDRG-based prognostic RS signature was constructed using The Cancer Genome Atlas as the training set and GSE39582 as the validation set. Two subsets with distinct differentiation states, involving 40 hub CDRGs regulated by YY1 and EGR2, were identified by single-cell RNA sequencing data, of which subset I was related to hypoxia, metabolic disorders, and inflammation, and subset II was associated with immune responses and ferroptosis. The CDRG-based molecular subtypes could successfully predict the clinical outcomes of the patients, the tumor microenvironment status, the immune infiltration status, and the potential response to immunotherapy and chemotherapy. A nomogram integrating a five-CDRG-based RS signature and prognostic clinicopathological characteristics could successfully predict overall survival, with strong predictive performance and high accuracy. The study emphasizes the relevance of CC cell differentiation for predicting the prognosis and therapeutic response of patients to immunotherapy and chemotherapy and proposes a promising direction for CC treatment and clinical decision-making.

## Introduction

Colon cancer (CC) is the third most frequently diagnosed malignant tumor worldwide, with 945,000 newly diagnosed cases per year, accounting for 9.4% of all cancer cases, and with 492,000 cancer deaths per year, accounting for 7.9% of total deaths (Parkin, 2001; Bray et al., 2018). According to the SEER database, the major histological types of CC are adenocarcinoma, mucinous adenocarcinoma, and signet ring cell carcinoma, which account for 90.1%, 8.7%, and 1.2% of CC cases, respectively, and the 5-year survival rates are 72.2%, 67.4%, and 41.4%, respectively. The clinical manifestations and prognosis of different pathological types vary greatly (Deng et al., 2006; Verhulst et al., 2012; Nitsche et al., 2013; Wang et al., 2016). Currently, primary treatment modalities for CC include chemotherapy along with surgery, radiotherapy, and, more recently, targeted therapy for patients with advanced late-stage CC, which significantly prolongs the overall survival (OS) and improves the prognosis of patients. However, the multidrug resistance of tumor cells to chemotherapy and molecular targeted therapy has been a major obstacle in cancer research, severely limiting the efficacy of CC therapy (Holohan et al., 2013; Li et al., 2013; Guo et al., 2017). Recently, new therapeutic methods such as immunotherapy have been gradually introduced into clinical practice (Pagès et al., 2018). Unfortunately, the OS of patients has not greatly improved, necessitating the development of novel strategies to overcome drug resistance in cancer therapy.

Intratumoral heterogeneity refers to the emergence of new gene mutations and molecular phenotypes in each filial generation after the malignant transformation of primary cells throughout continuous evolution. Therefore, a tumor is considered a mixture of different tumor cell clones (Dagogo-Jack and Shaw, 2018). Currently, intratumoral heterogeneity is a major cause of chemoresistance; therefore, it is essential to characterize the intratumoral heterogeneity of CC cells. The development of single-cell RNA sequencing (scRNA-seq) technology has provided an effective method to reveal the characteristics of the single-cell transcriptome, opening up new avenues to investigate intratumoral heterogeneity (Lovett, 2013; Liang et al., 2014). This study aimed to analyze the cell differentiation trajectory in CC through scRNA-seq and investigate its relationship with clinical outcomes and potential responses to immunotherapy and chemotherapy, to gain new insights into the development of treatment strategies against CC and the evaluation of patient prognoses.

## Materials and Methods

### Data Acquisition and Processing

The scRNA-seq data of 1,441 cells in 15 CC samples were downloaded from the GSE148345 dataset in the Gene Expression Omnibus (GEO, https://www.ncbi.nlm.nih.gov/geo/) database. The percentage of mitochondrial genes was calculated using the “PercentageFeatureSet” function in R software. The correlation between sequencing depth and length of mitochondrial gene sequences or total intracellular sequences was assessed by correlation analysis. Cells with an intracellular gene number <50, intracellular sequencing number <3, and mitochondrial gene fraction >25% were excluded. After data quality control, scRNA-seq data were normalized *via* the “LogNormalize” algorithm. Bulk RNA-seq and relevant clinicopathological data, such as sex, age, and grade, of 39 normal and 385 CC samples in The Cancer Genome Atlas (TCGA) database (http://cancergenome.nih.gov/) and 585 CC samples in the GSE39582 dataset were used for molecular typing identification and risk scoring (RS) signature generation. In the current study, CC samples with a survival time <30 days, ambiguous survival status, and unclear clinicopathological features were excluded. The clinicopathological data of each sample are shown in Table 1.

**TABLE 1:** Clinicopathological features of patients with colon cancer.

Variables	GSE39582 cohort (*n* = 585)	TCGA cohort (*n* = 385)
Age (year)		
Mean ± SD^a^	66.9 ± 13.2	67.0 ± 12.8
Sex		
Female	263 (45.0)	180 (46.8)
Male	322 (55.0)	205 (53.2)
Clinical stage		
I	38 (6.5)	66 (17.1)
II	271 (46.3)	151 (39.2)
III	210 (35.9)	103 (26.8)
IV	60 (10.3)	54 (14.0)
Unknown	6 (1.0)	11 (2.9)
T stage		
T1	12 (2.1)	10 (2.6)
T2	49 (8.4)	68 (17.7)
T3	379 (64.8)	263 (68.3)
T4	119 (20.3)	44 (11.4)
Unknown	26 (4.4)	0 (0.0)
N stage		
N0	314 (53.7)	231 (60.0)
N1	137 (23.4)	88 (22.9)
N2	100 (17.1)	66 (17.1)
N3	6 (1.0)	0 (0.0)
Unknown	28 (4.8)	0 (0.0)
M stage		
M0	499 (85.3)	286 (74.3)
M1	61 (10.4)	54 (14.0)
Unknown	25 (4.3)	45 (11.7)

a Standard deviation.

### Dimensionality Reduction and Cell Annotation

At a false discovery rate (FDR) <0.05, dimensions with significant separation were identified through principal component analysis (PCA) (Lall et al., 2018). Then, dimension reduction for the top 15 principal components (PCs) was performed using the t-distributed stochastic neighbor embedding (tSNE) algorithm to obtain the major clusters (Satija et al., 2015). Under the conditions of |log2[fold change (FC)]| > 0.5 and FDR <0.05, marker genes were screened out through differential expression analysis, and the top 10% of marker genes from clusters were displayed on a heat map. Clusters were annotated using the “SingleR” package in R and the CellMarker database (http://biocc.hrbmu.edu.cn/CellMarker/).

### Cell Trajectory Analysis and Molecular Functional Analysis

Pseudotime and cell differentiation trajectory were performed using the “Monocle” package in R (Qiu et al., 2017). With the criteria of |log2(FC)| > 1 and FDR <0.05, the differentially expressed genes identified from cell differentiation trajectories were designated CC differentiation-related genes (CDRGs). The “clusterProfiler” package in R was used for Kyoto Encyclopedia of Genes and Genomes (KEGG) enrichment analysis and Gene Ontology (GO) annotation for three domains, namely, biological process, cellular component, and molecular function. Hub CDRGs associated with cell differentiation trajectories were screened out through weighted correlation network analysis (WGCNA). Transcription factor (TF) enrichment analysis was conducted in the UCSC Xena platform (http://xena.ucsc.edu/).

### Identification of CDRG-Based Molecular Subtypes of Patients With CC From the GSE39582 Dataset

The “ConsensusClusterPlus” package in R was used for consensus clustering of CC samples after univariate analysis (Wilkerson and Hayes, 2010). The PCA and tSNE algorithms were used to verify the stability of CC subtypes. Kaplan–Meier analysis was conducted using the “survival” package in R. Clinicopathological features were displayed on histograms using the “ggplot2” package. Differentially expressed CDRGs with FDR <0.05 in specific molecular subtypes were screened for exploring their expression levels within different cell differentiation trajectories.

### Integrated Analysis of Tumor Microenvironment Status, Immune Infiltration, and Immune Checkpoint Gene Expression Across CC Subtypes

The immune/stromal scores and tumor purity of each sample were calculated using the “ESTIMATE” package in R. Single-sample gene set enrichment analysis (ssGSEA) was performed using the “GSVA” package to quantify the enrichment level of each sample in 29 immune gene sets (Supplementary Material S1). The fraction of 22 immune cells in each sample from the GSE39582 dataset was predicted using the CIBERSORT algorithm. Thirty-seven immune checkpoint genes (ICGs) were accessed through an extensive literature search (Wu et al., 2016; Nishino et al., 2017; Patel et al., 2017; Yang et al., 2017; Garris et al., 2018; Zhang et al., 2018; Wang et al., 2019a; Wang et al., 2019b; Han et al., 2019). Differential expression of genes across CC subtypes was assessed using the “limma” package in R.

### Prediction of Chemotherapeutic Response (IC50) for CC Subtypes

The “pRRophetic” package has been extensively used in cancer-related studies (PMID: 33115513, 33251044, 33738339, 34532269, 34335575, and 25229481), which can employ ridge regression to estimate the half-maximum inhibitory concentration (IC50) of samples. In this study, the “pRRophetic” package was used to predict the chemotherapeutic response of each sample in the GSE39582 dataset on the basis of the Genomics of Drug Sensitivity in Cancer (GDSC) database (https://www.cancerrxgene.org/). Twelve chemotherapeutic agents were selected, namely, camptothecin, mitomycin C, doxorubicin, gemcitabine, paclitaxel, rapamycin, sorafenib, bleomycin, docetaxel, sunitinib, cisplatin, and vinblastine, to determine the response of CC cells to chemotherapy. On the basis of the GDSC training set, 10-fold cross-validation was performed to evaluate the accuracy of the prediction of chemotherapy response.

### Construction and Validation of RS Signature

The bulk RNA-seq data were normalized with the log2 scale transformation method. Intersected CDRGs from TCGA and GSE39582 cohorts were identified using the “SVA” package in R. Differential expression analysis was performed in the TCGA cohort with |log2(FC)| > 1 and FDR <0.05. Univariate analysis was performed for obtaining prognostic CDRGs in the TCGA cohort. With TCGA as the training set and GSE39582 as the validation set, the prognostic CDRGs were incorporated into Lasso Cox regression analysis to construct a CDRG-based RS signature. The RS of each sample was calculated as the sum of the products of CDRGs expression levels and coefficients, using the following formula:

$$\text{RS}=\sum_{i}^{k}\left(Exp_{i}\times Coe_{i}\right),$$

where “*i*” and “*k*” represent the “*i*th” gene and gene number, respectively. Kaplan–Meier analysis was conducted to compare the OS between the high-risk group and the low-risk group. Receiver operating characteristic (ROC) curves were plotted to evaluate the predictive performance of the RS signature.

### Assessment of Expression Levels of CDRGs in the RS Signature

A total of 20 pairs of CC samples were collected from patients with CC admitted to the Renmin Hospital of Wuhan University. The study was approved by the Ethics Committee of Renmin Hospital of Wuhan University (no. WDRY 2019-K092), and an informed consent was obtained from all patients. Total RNA was extracted from CC patient tissues using TRIzol reagent (Invitrogen, Carlsbad, CA, United States), and complementary DNA was synthesized using the total RNA and a PrimeScript RT reagent kit (Takara). Quantitative PCR (qPCR) was performed using SYBR Green (Takara) on a CFX-96 instrument (Bio-Rad Laboratories, Inc., United States). The data were computed using the 2^−ΔΔCt^ method, and expression levels of target genes were normalized to those of *GAPDH*. The primer sequences used for qPCR in this study are listed in Table 2. The raw data of qPCR are shown in Supplementary Material S2. Subsequently, the expression levels of CDRGs at the protein level were verified using the Human Protein Atlas database (www.proteinatlas.org).

**TABLE 2:** List of primers.

Gene	Primer sequence (5′-3′)
ACAA2	Forward: AGA​CCC​CAG​CTC​TCA​CGA​TT
	Reverse: GGC​TCA​TGC​TTT​CGG​TTC​CT
SRI	Forward: GAG​ACT​TGC​CGG​CTT​ATG​GT
	Reverse: TTG​TCA​GGG​CCT​TCT​GCA​AT
UGT2A3	Forward: TGG​TGT​TTT​CTC​TGG​GGT​CAC
	Reverse: ACA​GCC​GAG​TAT​TGG​CTC​CT
KPNA2	Forward: GTG​ATG​GCT​CAG​TGT​TCC​GA
	Reverse: GTG​CAG​GAT​TCT​TGT​TGC​GG
MRPL37	Forward: AGA​GAA​CCA​AGA​CGA​GTG​CG
	Reverse: CAC​CAG​AAC​CAC​GGA​CTT​GA

### Construction and Validation of a Nomogram to Predict OS

Univariate analysis was performed in the training and validation sets to obtain prognostic variables, which were enrolled into a nomogram for predicting 3- and 5-year OS. Then, ROC and calibration curves were used to evaluate the predictive performance of the nomogram. Furthermore, the validation set was used for estimating the stability of the nomogram.

## Results

### Quality Control and Normalization of scRNA-Seq Data

**FIGURE 1:**
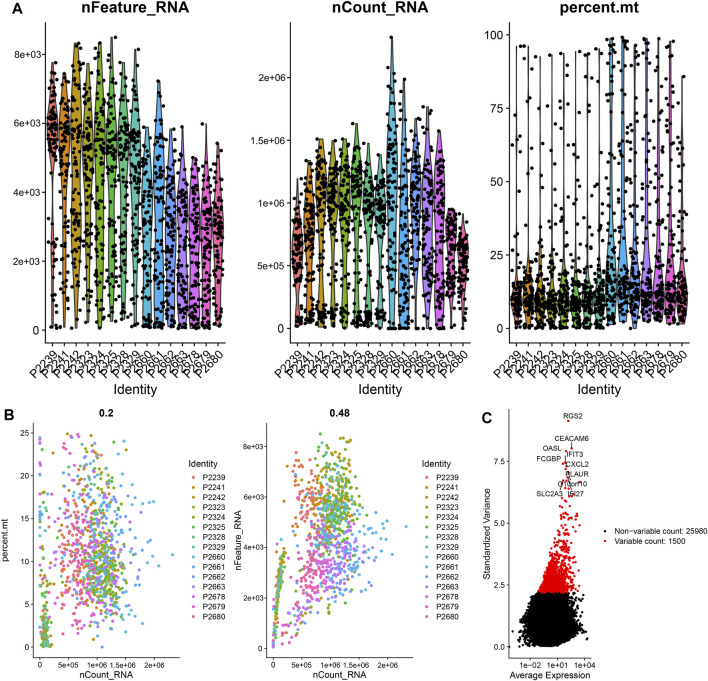
Quality control and normalization of scRNA-seq data. **(A)** After quality control, 1,092 CC cells were selected for analysis. **(B)** Results of correlation analysis. **(C)** The variation of 27,480 genes between CC cells. scRNA, single-cell RNA sequencing; CC, colon cancer.

The scRNA-seq data of 1441 CC cells in 15 samples were downloaded from the GSE148345 dataset. After performing quality control and normalization, 349 non-compliant cells were excluded, and 1,092 cells were selected for further analysis (Figure 1A). The correlation between sequencing depth and mitochondrial gene sequences was weak, with *R* = 0.2, which was the minimum correlation coefficient obtained according to the best screening conditions (Figure 1B). The sequencing depth was positively correlated with total intracellular sequences (*R* = 0.48, Figure 1B). A total of 27,480 genes were subjected to further analysis, of which 25,980 were intercellular genes with a low-mutation frequency, and 1,500 were genes with high variability (Figure 1C).

### Identification of Two CC Subsets by CC Cell Trajectory Analysis

**FIGURE 2:**
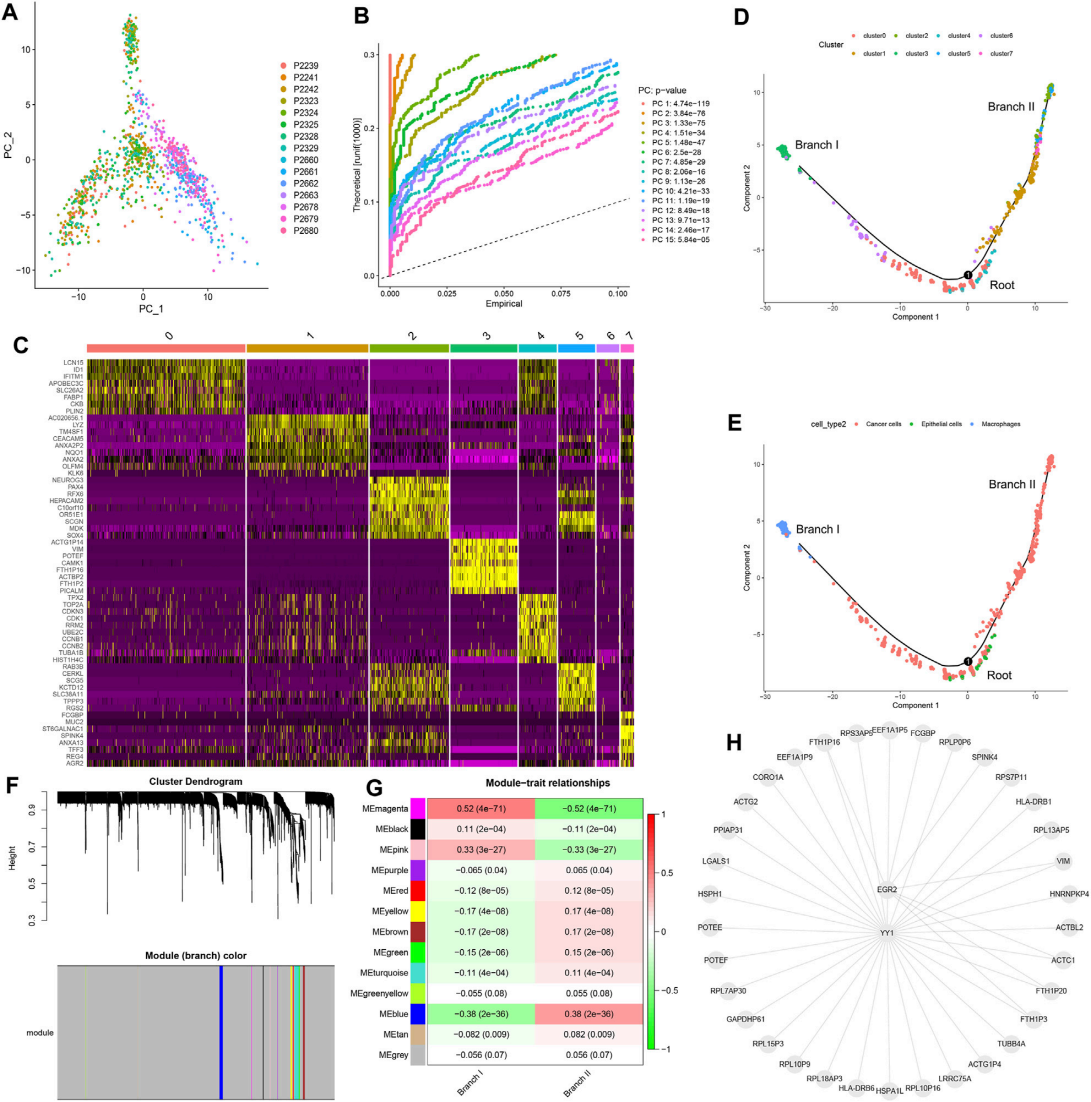
Cell trajectory analysis and WGCNA. **(A)** PCA based on scRNA-seq data. **(B)** The top 15 PCs. **(C)** Eight clusters were identified by tSNE algorithm, and a heat map was plotted to represent marker genes (top 10%) in each cluster. **(D)** Two subsets (namely, branch I and branch II) with distinct differentiation states were identified by cell trajectory analysis. **(E)** Cell types of subset I (branch I), subset II (branch II), and root. **(F)** Thirteen modules were identified using WGCNA. **(G)** Correlation analysis between modules and subsets. **(H)** Transcription factor enrichment analysis of key CDRGs. PCA, principal component analysis; PCs, principal components; tSNE, t-distributed stochastic neighbor embedding; CC, colon cancer; CDRG, CC differentiation-related gene. WGCNA, weighted correlation network analysis.

Preliminary dimensionality reduction of scRNA-seq data was performed by PCA, but no significant segregation was observed among CC cells (Figure 2A). Therefore, the top 15 PCs with significant differences were screened for further analysis (Figure 2B). According to the tSNE algorithm, 1,092 CC cells were clustered into eight clusters, 5,224 marker genes were identified, and the top 10% of marker genes into each cluster were displayed on the heat map (Figure 2C). Eight clusters were annotated on the basis of marker genes: clusters 0/1/2/5/6/7 were cancer cells, cluster 3 was macrophages, and cluster 4 was epithelial cells. The results of cell trajectory analysis revealed that cluster 4 was mainly distributed in the root, containing epithelial cells. Clusters 0/3/6 were distributed in subset I (branch I), which consisted of cancer cells and macrophages. Clusters 1/2/5/7 were located in subset II (branch II), which consisted of cancer cells (Figures 2D,E).

### Molecular Functional Analysis Based on CDRGs

We identified 153 CDRGs from the root, 800 CDRGs from subset I (branch I), and 245 CDRGs from subset II (branch II). The results of GO annotation and KEGG enrichment analysis revealed that overexpression of CDRGs in root was strictly related to the regulation of nuclear mitosis (Supplementary Figures S1A,B), and low expression of CDRGs was closely associated with immune killing (Supplementary Figures S1C,D). Highly expressed CDRGs in subset I (branch I) were steadily linked to hypoxia and inflammatory responses (Supplementary Figures S2A,B), and lowly expressed CDRGs were correlated with energy metabolic disorders (Supplementary Figures S2C,D). The upregulated CDRGs in subset II (branch II) were profoundly associated with cellular drug metabolism and immune responses (Supplementary Figures S2A,B), whereas the downregulated CDRGs were strongly correlated with ferroptosis and catabolism (Supplementary Figures S3C,D).

### Identification of Hub CDRGs in Two Subsets

On the basis of WGCNA, 13 modules were generated with soft threshold = 6 (Figure 2F), of which three modules were positively correlated with subset I (branch I), whereas nine modules were positively associated with subset II (branch II) (Figure 2G). In this study, the magenta module and the blue module were regarded as core modules that regulated cell differentiation trajectories, and hub CDRGs in two core modules are shown in Supplementary Material S3. The results of TF enrichment analysis showed that YY1 and EGR2 were the key TFs regulating the transcription of hub CDRGs (Figure 2H).

### Identification of CDRG-Based Molecular Subtypes of Patients With CC From the GSE39582 Dataset

**FIGURE 3:**
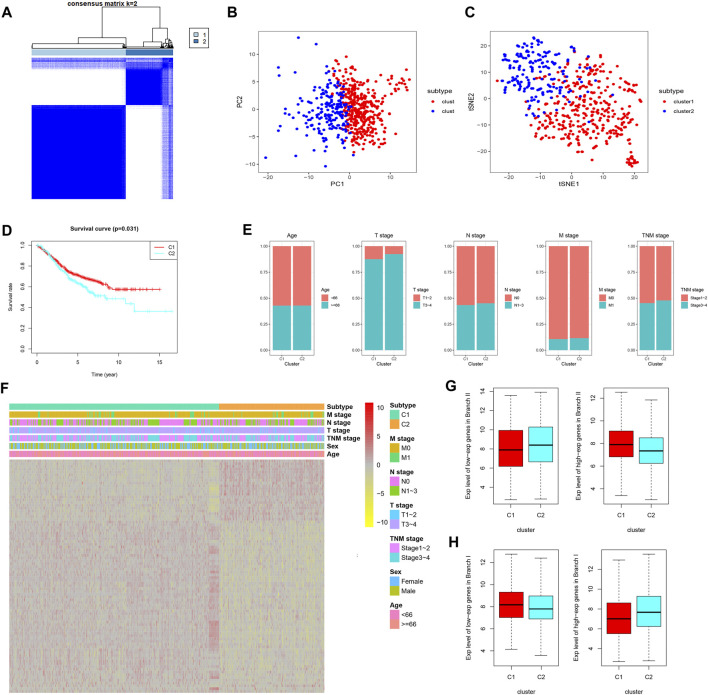
CDRG-based consensus clustering analysis of CC patients from the GSE39582 dataset. **(A–C)** Two molecular subtypes were identified at a clustering threshold of maxK = 2. **(B)** PCA. **(C)** tSNE algorithm. **(D)** Kaplan–Meier analysis between subtype 1 (C1) and subtype 2 (C2). **(E)** The proportion of clinicopathological variables across the two subtypes. **(F)** A heat map of differentially expressed CDRGs among the two subtypes. **(G)** The down/upregulated CDRGs in subtype 1 (C1) showed the same expression trend as that in subset II (branch II). **(H)** The down/upregulated CDRGs in subtype 2 (C1) showed the same expression trend as that in subset I (branch I). CC, colon cancer; CDRGs, CC differentiation-related genes; PCA, principal component analysis; tSNE, t-distributed stochastic neighbor embedding.

Prognostic CDRG-based consensus clustering analysis was performed on the GSE39582 dataset, and two molecular subtypes, including all the CC samples, were generated at a clustering threshold of *K* = 2 (Figure 3A). The PCA and tSNE algorithms demonstrated that the two clusters have strong stability (Figures 3B,C). The Kaplan–Meier method was used to determine the statistical significance of the consensus clustering results for CC, and the results revealed that subtype 1 (C1) had better OS compared with that of subtype 2 (C2) (Figure 3D, *p* = 0.031). Moreover, subtype 2 (C2) had more patients with a disease of advanced T stage and TNM stage (Figure 3E). Differentially expressed CDRGs between two molecular subtypes are displayed on a heat map (Figure 3F): The down/upregulated CDRGs in subtype 1 (C1) showed the same expression trend as that in subset II (branch II) (Figure 3G), whereas CDRGs in subtype 2 (C2) showed the same expression trend as that in subset I (branch I) (Figure 3H). These results demonstrated that subtype 1 (C1) was mainly composed of CC cells in subset II (branch II), and subtype 2 (C2) was composed of CC cells in subset I (branch I).

### Integrated Analysis of Tumor Microenvironment Status and Immune Infiltration Between Two Molecular Subtypes

**FIGURE 4:**
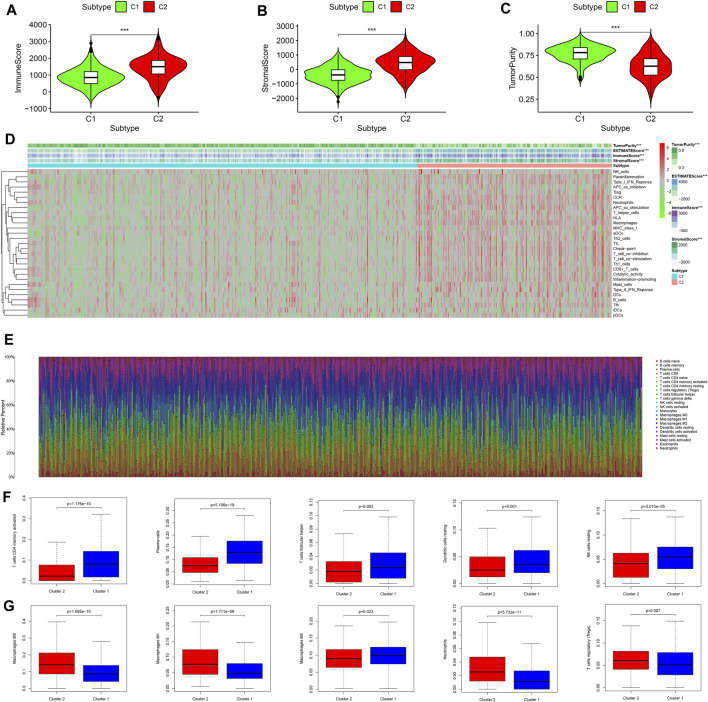
Comprehensive analysis of the tumor microenvironment status and immune infiltration across two molecular subtypes. **(A)** Immune scores of the two molecular subtypes. **(B)** Stromal scores of the two molecular subtypes. **(C)** Tumor purity status of the two molecular subtypes. **(D)** Heat map of ssGSEA scores for the two molecular subtypes. **(E)** The infiltration density of 22 immune cells in each sample. **(F)** Immune cells with higher fraction in subtype 1 (C1). **(G)** Immune cells with higher fraction in subtype 2 (C2). ssGSEA, single-sample gene set enrichment analysis.

On the basis of the “ESTIMATE” package, the immune/stromal scores (all *p* < 0.001) of subtype 2 (C2) were higher than those of subtype 1 (C1) and the tumor purity (*p* < 0.001) of subtype 2 (C2) was lower than that of subtype 1 (C1) (Figures 4A–C). The results of ssGSEA showed that the immune activity of subtype 2 (C2) was significantly higher than that of subtype 1 (C1) (Figure 4D), which was consistent with the immune score (Figure 4A). The infiltration density of 22 types of immune cells in each sample was calculated on the basis of the CIBERSORT algorithm, and the results were presented as different colors, which represented different cell types (Figure 4E). The results of differential analysis further demonstrated that subtype 1 (C1) contained a higher distribution of memory-activated CD4^+^ T cells, plasma cells, follicular T helper cells, dendritic cells, and natural killer (NK) cells (Figure 4F, all *p* < 0.05), whereas subtype 2 (C2) had a higher distribution of macrophages, neutrophils, and regulatory T cells (Tregs) (Figure 4G, all *p* < 0.05). Together, subtype 2 (C2) with higher immune infiltration was associated with tumor immune escape mediated by Tregs and macrophages. However, subtype 2 (C2) lacked cytotoxic T lymphocytes, which are involved in anti-tumor immunity.

### Comparison of Expression Levels of ICGs Between Two Molecular Subtypes

**FIGURE 5:**
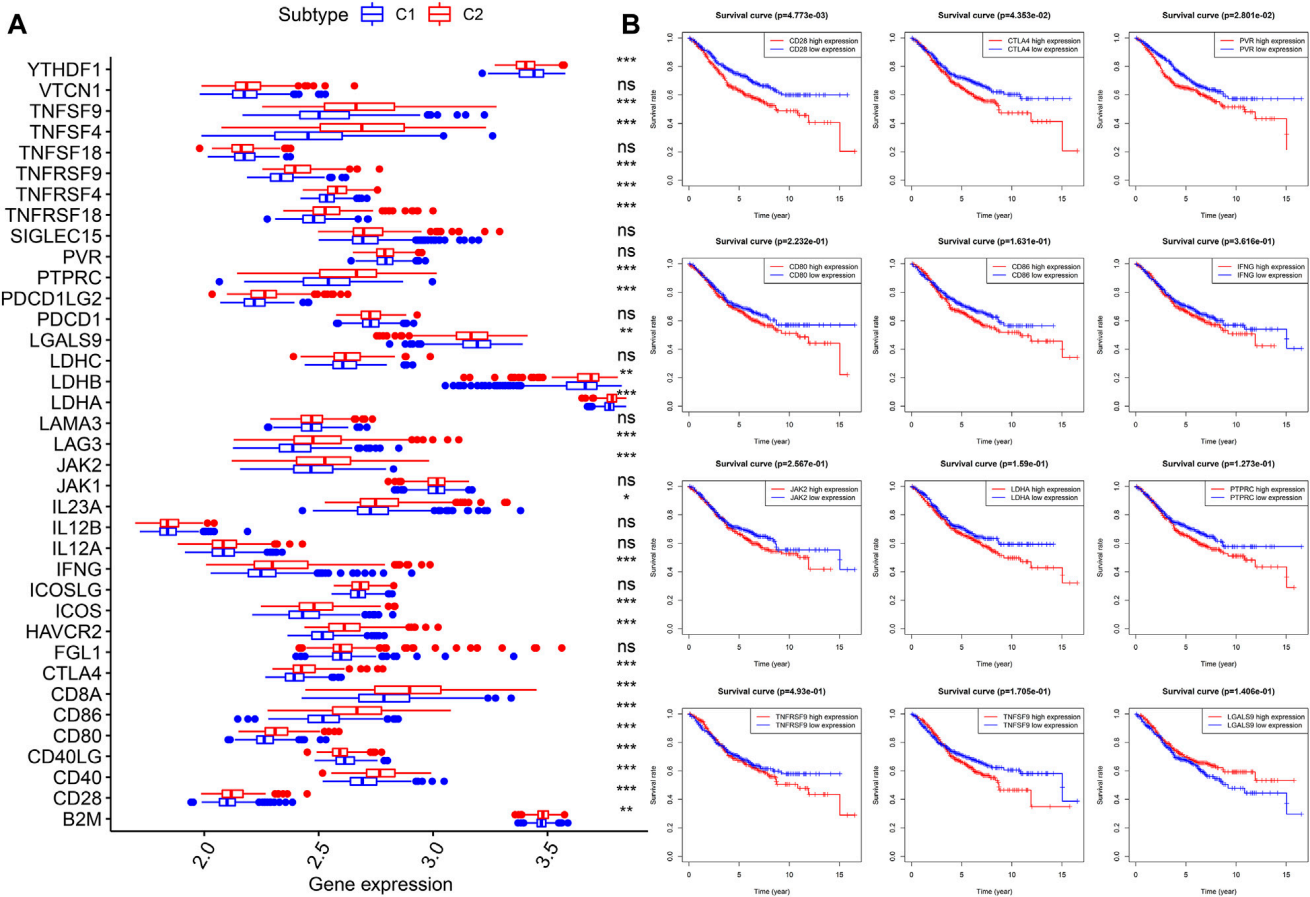
Expression levels and prognostic values of ICGs in two molecular subtypes. **(A)** Differential expression analysis of 37 ICGs. **(B)** Kaplan–Meier analysis of ICGs. ICGs, immune checkpoint genes.

Thirty-seven confirmed ICGs were accessed from previous studies (Wu et al., 2016; Nishino et al., 2017; Patel et al., 2017; Yang et al., 2017; Garris et al., 2018; Zhang et al., 2018; Wang et al., 2019a; Wang et al., 2019b; Han et al., 2019). Through differential expression analysis, we found 23 upregulated ICGs (*B2M*, *CD28*, *CD40*, *CD80*, *CD86*, *CD8A*, *CTLA4*, *HAVCR2*, *ICOS*, *IFNG*, *IL23A*, *JAK2*, *LAG3*, *LDHA*, *LDHB*, *PDCD1LG2*, *PTPRC*, *PVR*, *TNFRSF18*, *TNFRSF4*, *TNFRSF9*, *TNFSF4*, and *TNFSF9*) in subtype 2 (C2) (Figure 5A, all *p* < 0.05). More importantly, upregulated ICGs, including *CD28* (*p* < 0.05), *CTLA4* (*p* < 0.05), *PVR* (*p* < 0.05), *CD80*, *CD86*, *IFNG*, *JAK2*, *LDHA*, *PTPRC*, *TNFRSF9*, and *TNFSF*, were correlated with worse OS (Figure 5B). Only three ICGs (namely, *CD40LG*, *LGALS9*, and *YTHDF1*) were highly expressed in subtype 1 (C1) (Figure 5A, all *p* < 0.05). Interestingly, upregulated *LGALS9* was correlated with a better OS (Figure 5B), whereas *CD40LG* and *YTHDF1* were not associated with OS. These results indicate that upregulated ICGs are associated with a higher distribution of Tregs and adverse clinical outcomes of subtype 2 (C2) and are hence vital for guiding immunotherapy of CC subtypes.

### Differences in the Sensitivity of Two Molecular Subtypes to Chemotherapy

**FIGURE 6:**
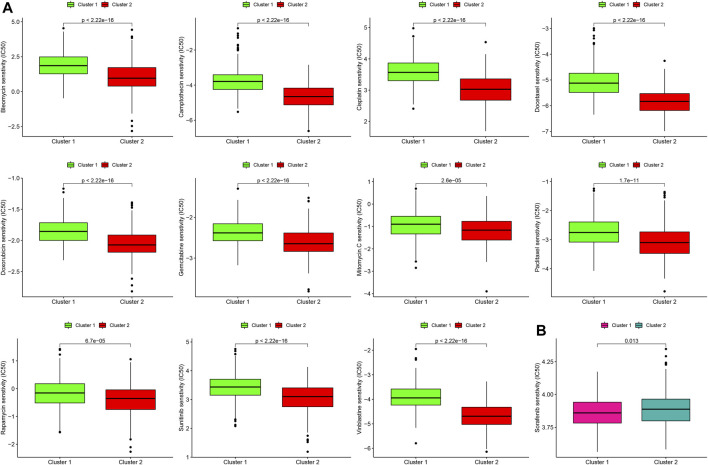
Differences in the sensitivity of two molecular subtypes to chemotherapy. **(A)** Chemotherapeutic agents that CC cells in subtype 2 (C2) are more sensitive to. **(B)** Chemotherapeutic agents that CC cells in subtype 1 (C1) are more sensitive to.

Next, we estimated the IC50 of 12 chemotherapeutic agents in each sample from the GSE39582 dataset. Subtype 2 (C2) was more sensitive to camptothecin, mitomycin C, doxorubicin, gemcitabine, paclitaxel, rapamycin, bleomycin, docetaxel, sunitinib, cisplatin, and vinblastine than subtype 1 (C1) (Figure 6A, all *p* < 0.001). However, subtype 1 (C1) was more sensitive to sorafenib than subtype 2 (C2) (Figure 6B, *p* = 0.013).

### Construction, Evaluation, and Validation of a Five-CDRG-Based RS Signature

**FIGURE 7:**
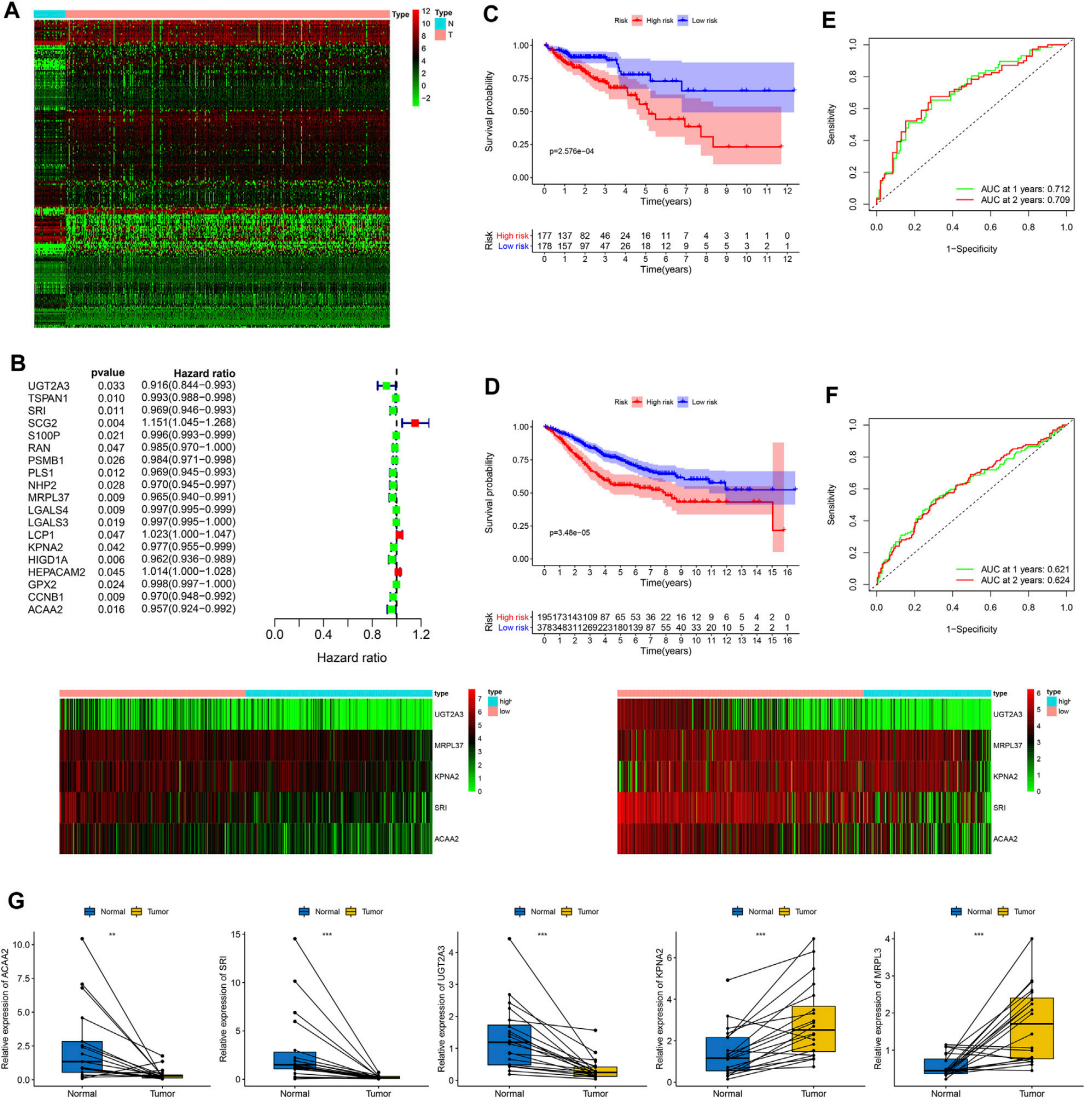
Generation of a prognostic RS signature and quantitative PCR for CDRGs. **(A)** Differential expression of 209 CDRGs in normal tissues and CC samples with |log2(FC)| > 1 and FDR <0.05. **(B)** Univariate analysis of CDRGs. Kaplan–Meier analysis between the low-risk group and the high-risk group in **(C)** the training set and **(D)** the validation set. The values of the areas under the ROC curves for predicting 3-, 4-, and 5-year OS in **(E)** the training set and **(F)** validation set. **(G)** Quantitative PCR for five CDRGs in the RS signature. *p < 0.05; **p < 0.01; ***p < 0.001. CC, colon cancer, CDRGs, CC differentiation-related genes, FDR, false discovery rate; OS, overall survival; ROC, receiver operating characteristic.

After obtaining the intersection of CDRGs in TCGA and GSE39582 datasets, a total of 673 CDRGs were subjected to differential analysis, and 209 differentially expressed CDRGs were identified (Figure 7A). The results of univariate analysis further revealed 19 prognostic CDRGs (Figure 7B), which were enrolled into Lasso Cox regression analysis, and finally, a five-CDRG-based RS signature was generated. The RS of each sample was calculated on the basis of the relative coefficient and expression levels of five genes using the following formula: RS= (−0.0427 * expression of *UGT2A3*) + (−0.0192 * expression of *SRI*) + (−0.0230 * expression of *MRPL37*) + (−0.0145 * expression of *KPNA2*) + (−0.0184 * expression of *ACAA2*). The RS of each CC sample was calculated in the training and validation cohorts, and the results revealed that the OS of the low-risk group was significantly better than that of the high-risk group (Figure 7C, TCGA: *p* = 2.576e-04; Figure 7D, GSE39582: *p* = 3.48e-05). Moreover, the values of the areas under the ROC curves for predicting 1- and 2-year OS were 0.712 and 0.709, respectively, in the training set (Figure 7E), and 0.621 and 0.624, respectively, in the validation set (Figure 7F).

### Expression Levels and Prognostic Values of Five CDRGs in the RS Signature

The results of qPCR analysis showed that *ACAA2* (*p* = 0.01), *SRI* (*p* < 0.001), and *UGT2A3* (*p* < 0.001) were downregulated in CC samples, whereas *KPNA2* (*p* < 0.001) and *MRPL37* (*p* < 0.001) were upregulated in CC samples (Figure 7G). The results of immunohistochemical staining confirmed that ACAA2 was highly expressed in normal glandular cells and moderately expressed in CC cells (Supplementary Figure S4A). KPNA2 was expressed at low intensity in normal glandular cells, endothelial cells, and stromal cells and was highly expressed in CC cells (Supplementary Figure S4B). Moreover, MRPL37 was moderately expressed in normal glandular cells and highly expressed in CC cells (Supplementary Figure S4C). SRI was upregulated in normal glandular cells and endothelial cells and was downregulated in CC cells (Supplementary Figure S4D). The expression of UGT2A3 was higher in normal glandular cells and endothelial cells and was significantly lower in CC cells than that in normal cells (Supplementary Figure S4E). The scRNA-seq data revealed that *ACAA2*, *KPNA2*, *MRPL37*, and *SRI* were downregulated in subset I (branch I, consisting of cluster 0/3/6), and *UGT2A3* was downregulated in subset II (branch II, containing cluster 1/2/5/7) (Supplementary Figures S4F,G). The results of Kaplan–Meier analysis demonstrated that upregulation of five CDRGs was correlated with better OS rates (Supplementary Figure S4H, all *p* < 0.05).

### Construction, Evaluation, and Validation of a Nomogram for Predicting OS

**FIGURE 8:**
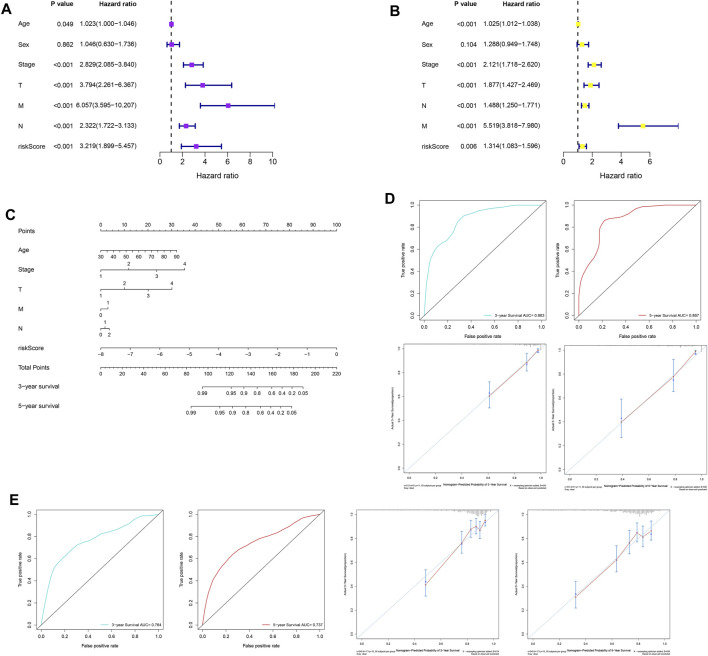
Construction, evaluation, and validation of a nomogram. Univariate analysis based on **(A)** the training set and **(B)** the validation set. **(C)** A nomogram was constructed on the basis of the training set. **(D)** In the training set, the areas under the ROC curves for predicting 3- and 5-year OS. **(E)** Calibration curves for predicting 3- and 5-year OS of patients in the training set. **(F)** The ROC curves and calibration curves for 3- and 5-year OS of patients in the validation set. ROC, receiver operating characteristic; OS, overall survival.

Univariate analysis was performed on the clinicopathological characteristics and RS in the training and validation cohorts, and the results revealed that patient age, TNM stage, T stage, N stage, M stage, and RS jointly affected patient prognosis (TCGA: Figure 8A, all *p* < 0.05; GSE39582: Figure 8B, all *p* < 0.05). Older age, later clinicopathological stage, and higher RS corresponded to poorer OS. Then, six prognostic factors were combined to establish a nomogram for predicting the OS of patients with CC on the basis of the training set (Figure 8C). The values of the areas under the ROC curves for predicting 3- and 5-year OS were 0.863 and 0.867, respectively (Figure 8D), and the calibration curves for predicting 3- and 5-year OS were in good agreement with the actual observations (Figure 8D). In addition, the nomogram with the strongest predictive performance was verified in the training set (Figure 8E).

## Discussion

Previous research has highlighted the importance of tumor site, pathological type, early or advanced stage, therapy phase, and primary and metastatic focus in evaluating the therapeutic response and prognosis of patients with CC (Deng et al., 2006; Verhulst et al., 2012; Nitsche et al., 2013; Wang et al., 2016). Given the complexity of CC heterogeneity, the relationship between CC heterogeneity and clinical outcome and treatment response needs to be further studied. In the present study, we identified two CC subsets with distinct differentiation states, of which subset I (branch I) was related to hypoxia, metabolic disorders, and inflammatory responses, and subset II (branch II) was associated with immune responses and ferroptosis. CDRG-based consensus molecular classifications successfully predicted clinical outcomes, immune infiltration status, potential immunotherapy, and chemotherapeutic response in patients with CC. A nomogram combining the five-CDRG-based RS signature and prognostic clinicopathological characteristics accurately predicted the OS of patients with CC.

Gene-dependent intratumoral heterogeneity is an important factor that determines the biological behavior of CC cells. Different pathogeneses, natural evolution, and medical interventions produce a large number of molecular phenotypes, which can lead to differences in clinical features, therapeutic response, drug resistance, and prognosis among individuals with CC (Hanahan and Weinberg, 2011; Dagogo-Jack and Shaw, 2018). In this study, CC cells with distinct differentiation states were projected into two subsets on the basis of cell trajectory analysis. These results demonstrate that CC cell differentiation may be related to the activation/inhibition of various signaling pathways in a time- and dose-dependent manner. On the basis of WGCNA and TF enrichment analysis, we provided a benchmark for the investigation of intratumoral heterogeneity and the recognition of specific molecular phenotypes in CC.

Different molecular phenotypes and molecular types can guide disease diagnosis, optimize therapeutic strategies, and facilitate the development of new models for the advancement of precision medicine. In current studies, CC has been classified according to gene mutations, copy number, promoter methylation, non-coding RNA, microsatellite instability, and proteomics, and molecular subtypes of CC are based on tumor characteristics, therapeutic response, and prognosis, with an inconsistent pattern (Cancer Genome Atlas Network, 2012; Schlicker et al., 2012; Sinicrope and Sargent, 2012; Budinska et al., 2013; De Sousa E Melo et al., 2013; Marisa et al., 2013; Sadanandam et al., 2013; Roepman et al., 2014; Guinney et al., 2015). Therefore, it is worth exploring the possibility of introducing individualized treatment of CC based on molecular subtyping. In this study, CDRG-based molecular typing showed that the OS of subtype 1 (C1) was superior to that of subtype 2 (C2). Previous studies have shown that the infiltration of lymphocytes, such as CD4^+^ T cells, NK cells, and plasma cells, was high in the tumor microenvironment (Almatroodi et al., 2016; Engblom et al., 2016; Jiang et al., 2016; Jain et al., 2017; Janakiram et al., 2017; Pierce et al., 2017; Song et al., 2017) and that the effectiveness of immunotherapy may be attributed to the upregulation of ICGs (e.g., programmed death-1, programmed death ligand-1, and cytotoxic T lymphocyte antigen-4) (Llosa et al., 2015). The infiltration density of memory-activated CD4^+^ T cells, follicular T helper cells, and NK cells was higher in subtype 1 (C1) than in subtype 2 (C2), and the proportion of macrophages and Tregs was lower in subtype 1 (C1) than in subtype 2 (C2). Moreover, the proportion of ICGs was higher in subtype 2 (C2) than in subtype C1 (C1), suggesting that the subtype C2 (C2) tumors are characterized by immune escape and immunosuppression. Nevertheless, immune checkpoint inhibitors may reactivate the immune response of subtype 2 (C2) to exert anti-tumor effects (Riaz et al., 2017). Furthermore, patients with subtype 2 (C2) can be treated with several chemotherapeutic agents for improved prognosis.

The results of molecular subtyping revealed that patients with subtype 1 (C1) had better survival outcomes, suggesting that the classification based on CDRGs can be used to predict the OS of patients with CC. Therefore, a CDRG-based RS signature with high accuracy and efficiency was established to evaluate the survival risk of patients with CC. To our knowledge, this is the first CDRG-based RS signature generated using Lasso Cox regression analysis, which can identify the gene combination with the best predictive ability. Moreover, a nomogram combining the CDRG-based RS and prognostic clinicopathological variables served as a visual model to predict patient OS, which was more accurate and effective than the RS signature.

There are some limitations to the current study. First, the prognostic RS signature was generated and verified on the basis of retrospective data from public open databases. Large-scale prospective clinical studies are needed to estimate the effectiveness and practicability of the RS signature. Second, although the nomogram for predicting OS has high predictive power, some clinicopathological parameters associated with prognosis are not available from the public open database, resulting in a limited number of variables involved in the nomogram. Therefore, it is necessary to further improve the nomogram in terms of higher predictive power and inclusion of more variables.

## Conclusion

In this study, we identified two subsets with distinct differentiation states and revealed that CC molecular subtypes on the basis of cell differentiation trajectories could successfully predict the patient prognosis, tumor microenvironment status, immune cell infiltration, and potential response to immunotherapy and chemotherapy. Moreover, the nomogram combining CDRG-based RS signature and clinicopathological characteristics accurately predicted the OS of patients with CC. Together, this study emphasizes the implications of CC cell differentiation for predicting prognosis and therapeutic response to immunotherapy and chemotherapy in patients and proposes a promising direction for clinical decision-making regarding CC treatment and prognosis.

## Data Availability

The scRNA-seq data were accessed from the GSE148345 dataset (https://www.ncbi.nlm.nih.gov/geo/). The bulk RNA-seq data were obtained from the GSE39582 dataset (https://www.ncbi.nlm.nih.gov/geo/) and TCGA database (http://cancergenome.nih.gov/).

## Abbreviations

CC, colon cancer (CC); CDRGs, CC differentiation-related genes; FDR, false discovery rate; GDSC, genomics of drug sensitivity in cancer; GEO, Gene Expression Omnibus; GO, Gene Ontology; ICG, immune checkpoint gene; KEGG, Kyoto Encyclopedia of Genes and Genomes; OS, overall survival; PCA, principal component analysis; PC, principal component; ROC, receiver operating characteristic; RS, risk scoring; scRNA-seq, single-cell RNA sequencing; ssGSEA, single-sample gene set enrichment analysis; TCGA, the cancer genome atlas; TF, transcription factor; tSNE, t-distributed stochastic neighbor embedding; WGCNA, weighted correlation network analysis.
