# Supplementary figures and images for: Identification of Subtypes and a Prognostic Gene Signature in Colon Cancer Using Cell Differentiation Trajectories
Source: Front Cell Dev Biol. 2021 Dec 13;9:705537. doi: 10.3389/fcell.2021.705537 (PMC8710730; doi:10.3389/fcell.2021.705537)

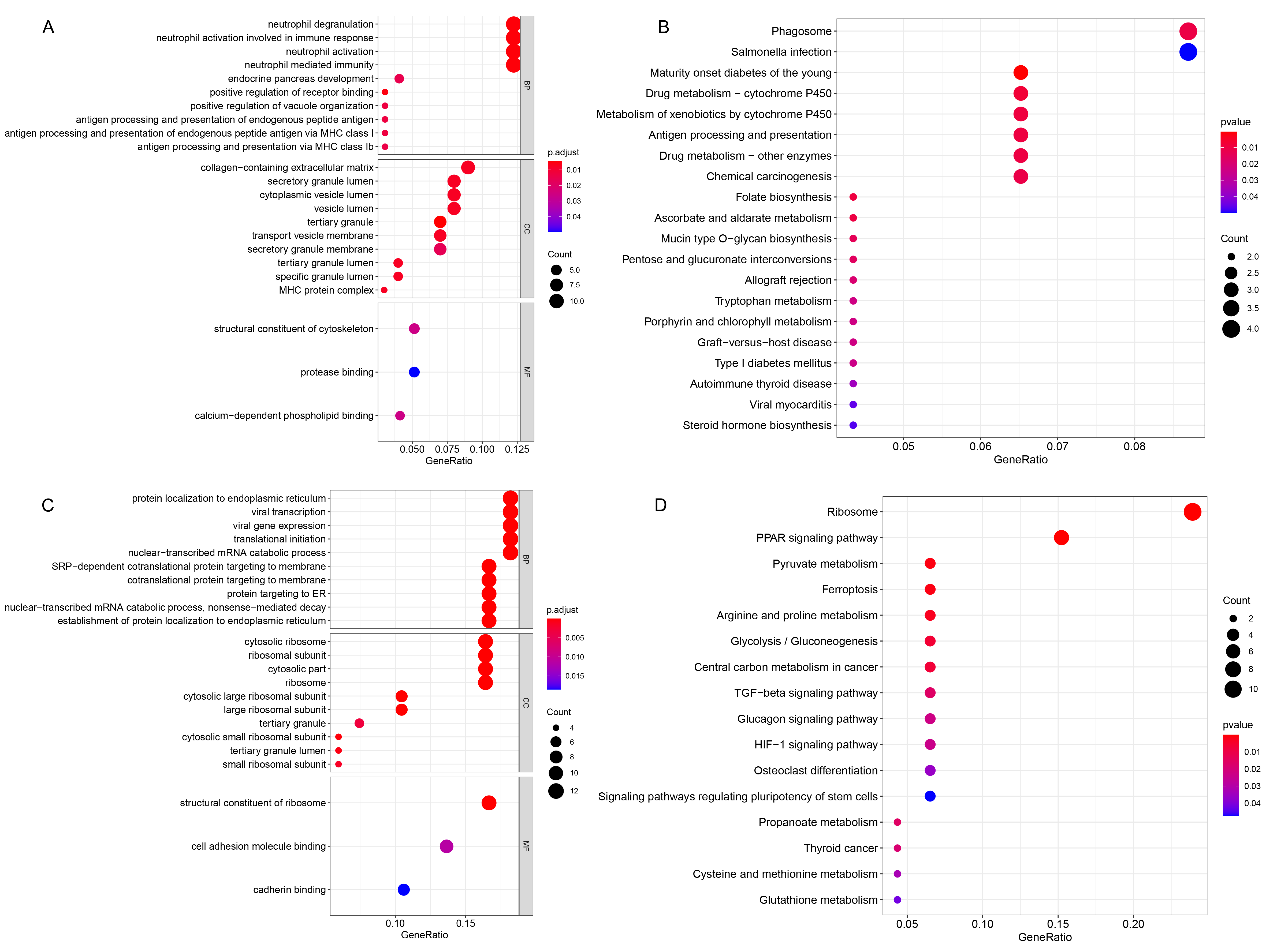

Supplement: Supplementary file 2 [file Image3.TIF]

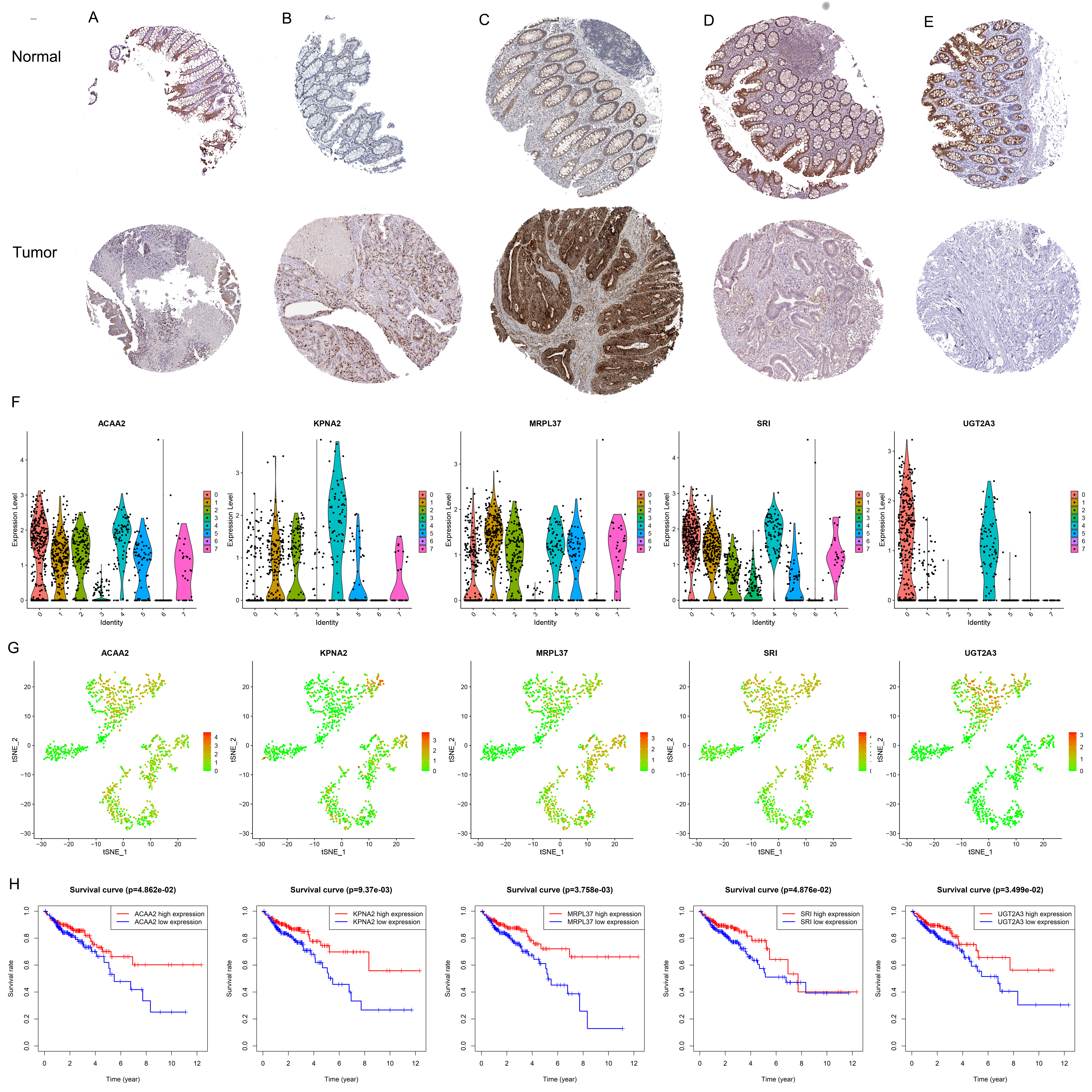

Supplement: Supplementary file 3 [file Image4.TIF]

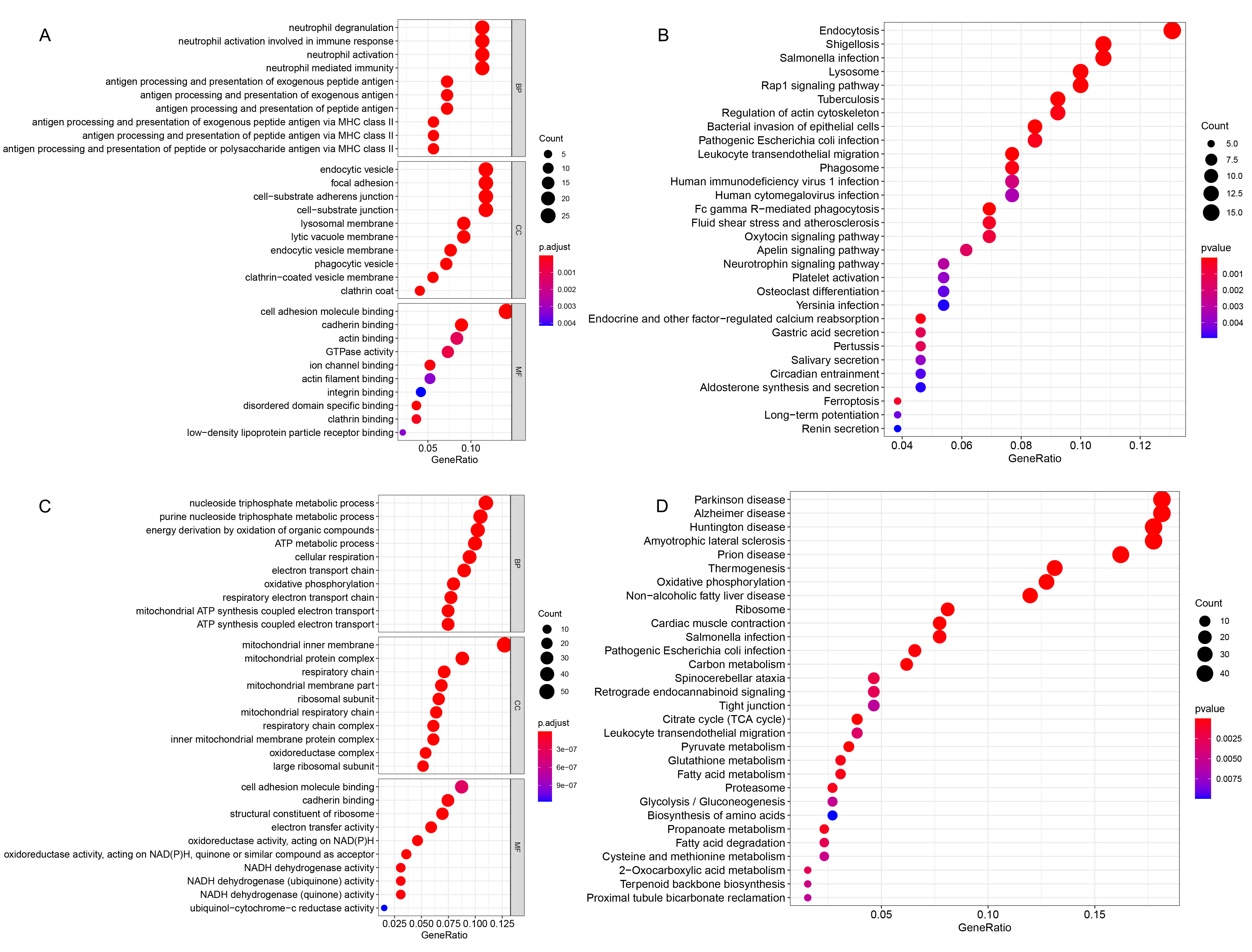

Supplement: Supplementary file 5 [file Image2.TIF]

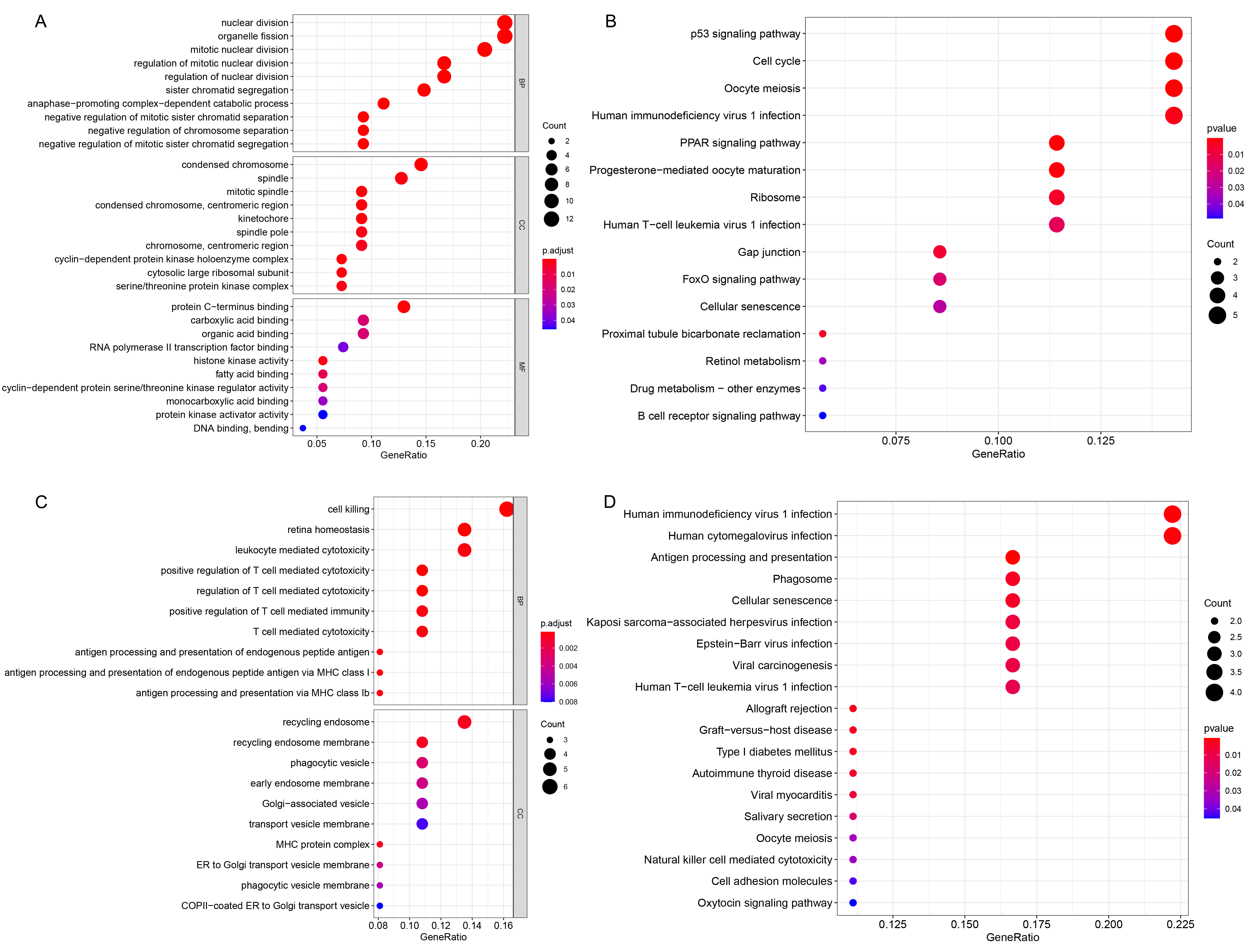

Supplement: Supplementary file 7 [file Image1.TIF]
