# Supplementary material for: Identification of Subtypes and a Prognostic Gene Signature in Colon Cancer Using Cell Differentiation Trajectories
Source: Front Cell Dev Biol. 2021 Dec 13;9:705537. doi: 10.3389/fcell.2021.705537 (PMC8710730; doi:10.3389/fcell.2021.705537)
Supplement: Supplementary file 8 [file Table1.DOC]

**Table 1. Clinicopathological features of patients with colon cancer.**

| **Variables** | **GSE39582 cohort (n=585)** | **TCGA cohort (n=385)** |
| --- | --- | --- |
| **Age (year)** | | |
| Mean ± SD* | 66.9 ± 13.2 | 66.9 ± 13.2 |
| **Sex** | | |
| Female | 263 (45.0) | 180 (46.8) |
| Male | 322 (55.0) | 205 (53.2) |
| **Clinical stage** | | |
| I | 38 (6.5) | 66 (17.1) |
| II | 271 (46.3) | 151 (39.2) |
| III | 210 (35.9) | 103 (26.8) |
| IV | 60 (10.3) | 54 (14.0) |
| Unknown | 6 (1.0) | 11 (2.9) |
| **T stage** | | |
| T1 | 12 (2.1) | 10 (2.6) |
| T2 | 49 (8.4) | 68 (17.7) |
| T3 | 379 (64.8) | 263 (68.3) |
| T4 | 119 (20.3) | 44 (11.4) |
| Unknown | 26 (4.4) | 0 (0.0) |
| **N stage** | | |
| N0 | 314 (53.7) | 231 (60.0) |
| N1 | 137 (23.4) | 88 (22.9) |
| N2 | 100 (17.1) | 66 (17.1) |
| N3 | 6 (1.0) | 0 (0.0) |
| Unknown | 28 (4.8) | 0 (0.0) |
| **M stage** | | |
| M0 | 499 (85.3) | 286 (74.3) |
| M1 | 61 (10.4) | 54 (14.0) |
| Unknown | 25 (4.3) | 45 (11.7) |

*standard deviation.
