# Supplementary material for: Identification of Subtypes and a Prognostic Gene Signature in Colon Cancer Using Cell Differentiation Trajectories
Source: Front Cell Dev Biol. 2021 Dec 13;9:705537. doi: 10.3389/fcell.2021.705537 (PMC8710730; doi:10.3389/fcell.2021.705537)
Supplement: Supplementary file 9 [file Table2.DOC]

**Table 2.** List of primers.

| **Gene** | **Primer sequence (5’-3’)** |
| --- | --- |
| ACAA2 | Forward: AGACCCCAGCTCTCACGATT |
|  | Reverse: GGCTCATGCTTTCGGTTCCT |
| SRI | Forward: GAGACTTGCCGGCTTATGGT |
|  | Reverse: TTGTCAGGGCCTTCTGCAAT |
| UGT2A3 | Forward: TGGTGTTTTCTCTGGGGTCAC |
|  | Reverse: ACAGCCGAGTATTGGCTCCT |
| KPNA2 | Forward: GTGATGGCTCAGTGTTCCGA |
|  | Reverse: GTGCAGGATTCTTGTTGCGG |
| MRPL37 | Forward: AGAGAACCAAGACGAGTGCG |
|  | Reverse: CACCAGAACCACGGACTTGA |
